# HTLV-1 Tax-1 interacts with SNX27 to regulate cellular localization of the HTLV-1 receptor molecule, GLUT1

**DOI:** 10.1371/journal.pone.0214059

**Published:** 2019-03-21

**Authors:** Jacob Al-Saleem, Wessel P. Dirksen, Michael P. Martinez, Nikoloz Shkriabai, Mamuka Kvaratskhelia, Lee Ratner, Patrick L. Green

**Affiliations:** 1 Center for Retrovirus Research, The Ohio State University, Columbus, Ohio, United States of America; 2 Department of Veterinary Biosciences, The Ohio State University, Columbus, Ohio, United States of America; 3 Division of Infectious Diseases, School of Medicine, University of Colorado Denver, Aurora, Colorado, United States of America; 4 Division of Oncology, Washington University, St Louis, Missouri, United States of America; 5 Comprehensive Cancer Center and Solove Research Institute, The Ohio State University, Columbus, Ohio, United States of America; Duke University Medical Center, UNITED STATES

## Abstract

An estimated 10–20 million people worldwide are infected with human T cell leukemia virus type 1 (HTLV-1), with endemic areas of infection in Japan, Australia, the Caribbean, and Africa. HTLV-1 is the causative agent of adult T cell leukemia (ATL) and HTLV-1 associated myopathy/tropic spastic paraparesis (HAM/TSP). HTLV-1 expresses several regulatory and accessory genes that function at different stages of the virus life cycle. The regulatory gene Tax-1 is required for efficient virus replication, as it drives transcription of viral gene products, and has also been demonstrated to play a key role in the pathogenesis of the virus. Several studies have identified a PDZ binding motif (PBM) at the carboxyl terminus of Tax-1 and demonstrated the importance of this domain for HTLV-1 induced cellular transformation. Using a mass spectrometry-based proteomics approach we identified sorting nexin 27 (SNX27) as a novel interacting partner of Tax-1. Further, we demonstrated that their interaction is mediated by the Tax-1 PBM and SNX27 PDZ domains. SNX27 has been shown to promote the plasma membrane localization of glucose transport 1 (GLUT1), one of the receptor molecules of the HTLV-1 virus, and the receptor molecule required for HTLV-1 fusion and entry. We postulated that Tax-1 alters GLUT1 localization via its interaction with SNX27. We demonstrate that over expression of Tax-1 in cells causes a reduction of GLUT1 on the plasma membrane. Furthermore, we show that knockdown of SNX27 results in increased virion release and decreased HTLV-1 infectivity. Collectively, we demonstrate the first known mechanism by which HTLV-1 regulates a receptor molecule post-infection.

## Introduction

HTLV-1 was the first discovered human retrovirus [1]. It is estimated that 10–20 million people are currently infected with HTLV-1 worldwide, with endemic areas of infection in Japan, the Caribbean Islands, Central America, South America, and Africa [1–3]. HTLV-1 is the causative agent of an aggressive malignancy of CD4^+^ T cells known as adult T cell leukemia (ATL), and a neurological disorder known as HTLV-1 associated myelopathy/tropic spastic paraparesis (HAM/TSP) [1–3]. While most individuals infected with HTLV-1 remain clinically asymptomatic, around 5–10% of infected individuals develop HTLV-1 associated disease [4]. ATL develops up to three and four decades post-infection primarily in individuals infected in infancy, and the aggressive classifications of ATL have a less than six month median survival time post diagnosis [5,6]. HTLV-2, a closely related virus, is not associated with any diseases in humans [7]. The severity of the HTLV-1 associated diseases necessitates a better understanding of how HTLV-1 infects and transforms cells [8].

HTLV-1 is a delta-retrovirus that expresses several regulatory and accessory genes, including the regulatory protein Tax-1 [9]. Tax-1 is important for the HTLV-1 life cycle via its ability to recruit CREB and p300 to the viral promoter, resulting in increased viral gene transcription [10–12]. Tax-1 has also been shown to contribute to the oncogenic potential of HTLV-1. Tax-1 expression in transgenic mice leads to a leukemia/lymphoma like disease, while over expression of Tax-1 in the CTLL-2 cell line promotes IL-2 independent growth [13–16].

Previous studies have identified a PDZ binding motif (PBM) at the carboxyl-terminus of Tax-1, and demonstrated the importance of this domain for the transformation capabilities of Tax-1 [16,17]. Interestingly, this domain is not present on the HTLV-2 homolog, Tax-2 [17]. We postulated that the Tax-1 PBM domain facilitates interactions with cellular proteins important for the transforming capacity of Tax-1 and could explain the difference in pathogenesis between HTLV-1 and HTLV-2. We performed a mass spectrometry-based proteomics screen utilizing wild type Tax-1 and Tax-1 lacking a PBM (Tax-1 ΔPBM) to identify interactions mediated by this domain. We identified a novel Tax-1 interacting protein, sorting nexin 27 (SNX27), which interacted with wild type Tax-1 but not Tax-1 ΔPBM. The sorting nexin family of proteins is involved in endocytosis, endosomal sorting, and endosomal signaling [18]. SNX27 is a unique member of the sorting nexin family as it features a PDZ domain [19]. SNX27 uses the PDZ domain to bind to specific cargos, such as GLUT1, to facilitate their retrieval from endosomal compartments and recycling back to the plasma membrane or cell surface [19,20]. This recycling prevents these proteins from being degraded in the lysosome [19,20]. Previous studies have shown that knockdown of SNX27 results in a drastic redistribution of GLUT1, from the plasma membrane to the lysosome where it is degraded [20,21].

GLUT1 facilitates the transport of glucose across the plasma membrane of the cell where it is utilized for cellular metabolism [22]. GLUT1 also serves an important role in HTLV-1 biology as one of the three receptor molecules for HTLV-1. Neuropilin 1 (NRP-1) and heparan sulfate proteoglycans (HSPG) are the other two receptor molecules, and are involved specifically in the binding of HTLV-1 to target cells [23–25]. GLUT1 is the HTLV-1 receptor molecule involved in the fusion and entry of the virus to the target cell. It has been demonstrated that GLUT1 expression levels in target cells is directly correlated to infectivity [26]. A recent study demonstrated that over-expression of GLUT1 in cells producing HTLV-1 virus like particles (VLPs) decreased infectivity, while over-expression of GLUT3, a closely related protein, had no effect [27]. This study also demonstrated that reduction of GLUT1 levels in HTLV-1 producing cells increased infectivity. Other retroviruses, such as HIV-1, have developed mechanisms to promote the removal of receptor molecules from the surface of infected cells [28]. These functions aid the virus in two ways; first it lowers the possibility of a superinfection event occurring, and second, the surface expression of receptor molecules can inhibit viral budding and release [29]. Interestingly, HTLV-1 has no known mechanism for regulating the localization of its receptor molecules. We set out to determine whether the Tax-1 interaction with SNX27 allows HTLV-1 to regulate the localization of GLUT1, and what effect this may have on HTLV-1 infectivity. We found that Tax-1 expression did cause a change in GLUT1 localization similar to SNX27 knockdowns. Also, we observed that SNX27 knockdown in virus producing cells increased virus release but, surprisingly, lowered HTLV-1 infectivity.

## Results

### Identification of Tax-1 and Tax-1 ΔPBM cellular binding proteins in HEK293T cells

**Fig 1:**
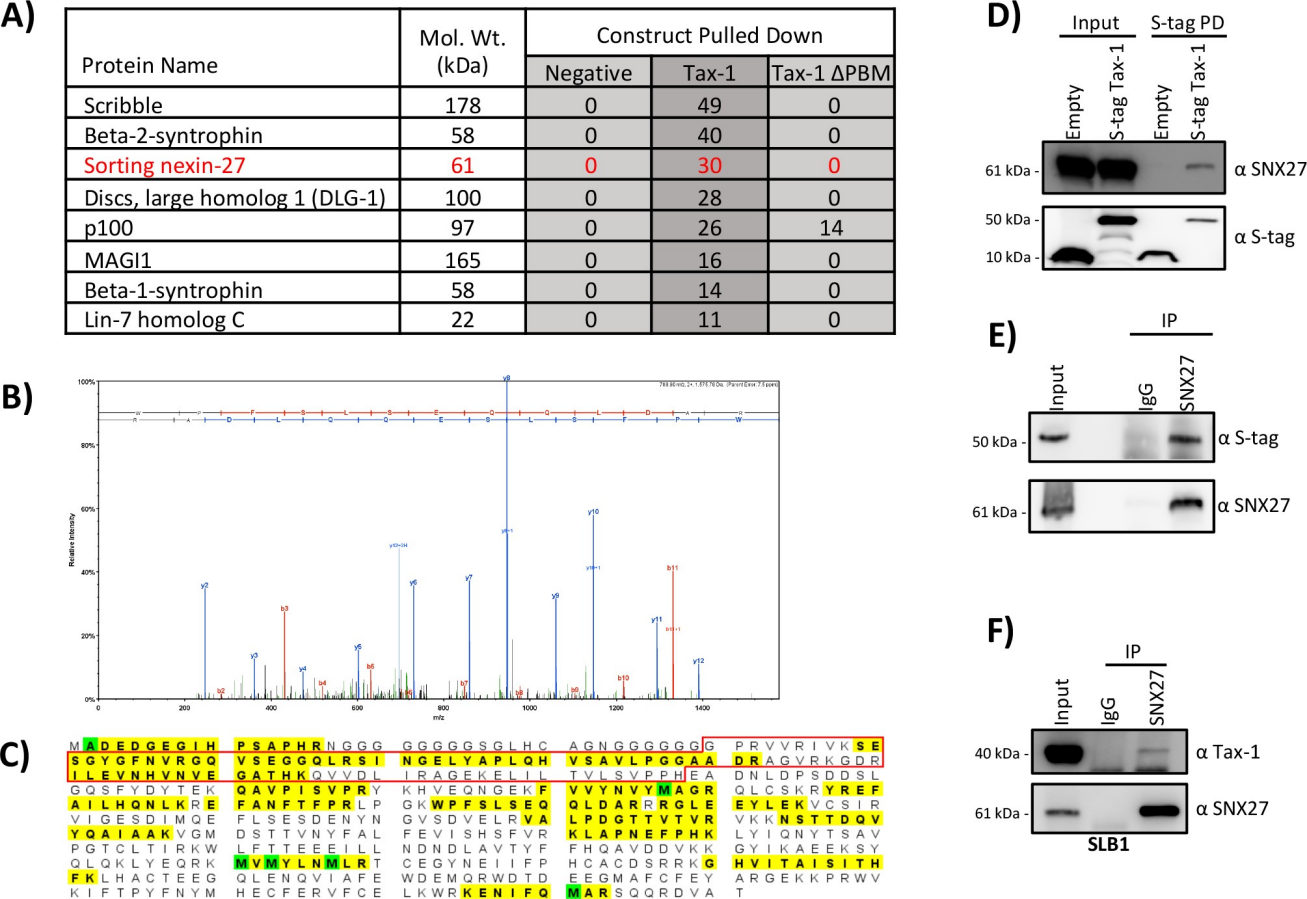
Identification of SNX27 as an Interaction Partner of Tax-1. **A)** Mass spectrometry results from S-tag pull-downs in HEK293T cells. The proteins listed on the left were identified in our data set. The total peptide counts for each interacting partner are indicated. A new binding partner of Tax-1, SNX27, is listed in red. **B)** Representative MS/MS Fragmentation of a SNX27 peptide. **C)** Schematic of peptide sequence of SNX27. The protein segments highlighted in yellow were detected by MS/MS. Modified residues are indicated in green. 27 unique spectra and 37 total spectra covered 204/541 amino acids (38% coverage) of SNX27. The red box indicates the peptides composing the SNX27 PDZ domain. **D)** Pull down demonstrating interaction between SNX27 and Tax-1. HEK293T cells were transfected with indicated S-tag constructs and 24 hours post transfection S-tag pull-downs were performed. Samples were subjected to SDS-PAGE followed by immunoblotting and probed for SNX27 and S-tag as shown. **E)** Reciprocal co-IP demonstrating interaction between SNX27 and Tax-1. HEK293T cells were transfected with S-tag Tax-1 and 24 hours later samples were subjected to co-IP using either SNX27 or normal IgG antibodies. Samples were subjected to SDS-PAGE followed by immunoblotting and probed for SNX27 and S-tag as shown. **F)** Co-IP demonstrating interaction between SNX27 and Tax-1 at endogenous levels of protein expression. SLB-1 cells were collected and lysed. Lysates were subjected to co-IP using either SNX27 or normal IgG antibodies. Samples were subjected to SDS-PAGE followed by immunoblotting and probed for SNX27 and S-tag, as shown.

To identify Tax-1 binding partners which required the PBM domain for interaction, we utilized a mass spectrometry-based proteomics approach. We compared proteins from HEK293T cellular lysates that interacted with the S-tag Tax-1, S-tag Tax-1 ΔPBM, and an empty S-tag expression vector as a negative control. The top proteins hits that were detected in S-tag Tax-1 fraction, but not in the negative control, are listed in Fig 1A. Our data contained several known interacting partners of Tax-1, including: the alternative NF-κB precursor protein p100, Scribble, Beta-1 and Beta-2-syntrophin, DLG-1, MAGI1, and Lin-7 homolog C [30–36]. The presence of these interactions demonstrated the robustness of our experimental approach and data.

We also uncovered a previously unknown interaction of WT Tax-1 with SNX27. SNX27 was selectively detected when Tax-1 was pulled down, but not when Tax-1 ΔPBM was pulled down (Fig 1A, 1B and 1C). Consistent with the mass spectrometry results, pull-down assays followed by immunoblotting showed that WT Tax-1, but not the negative control empty S-tag, interacted with SNX27 (Fig 1D). For further confirmation of the interaction, we followed the pull-down assay with a reciprocal co-immunoprecipitation (co-IP) assay for SNX27. In Tax-1 transfected HEK293T cells, SNX27 was immunoprecipitated and Tax-1 was found to co-precipitate, while no SNX27 or Tax-1 were detected in the IgG control sample (Fig 1E). We next investigated whether the interaction between Tax-1 and SNX27 could be detected in the absence of exogenous over expression of Tax-1 or SNX27. We utilized the HTLV-1 transformed CD4^+^ T cell line SLB-1, which expresses endogenous Tax-1, in a co-IP using SNX27 antibody [37]. Probing for SNX27 revealed efficient IP of SNX27, while probing with Tax-1 antisera demonstrated that Tax-1 co-precipitated with SNX27 in the absence of exogenous expression (Fig 1F). Collectively, these results confirmed the interaction between viral Tax-1 and cellular SNX27; moreover, this interaction was detected in HTLV-1 infected cells without exogenous over expression of either protein.

### Tax-1 and SNX27 interact via their PBM and PDZ domains, respectively

**Fig 2:**
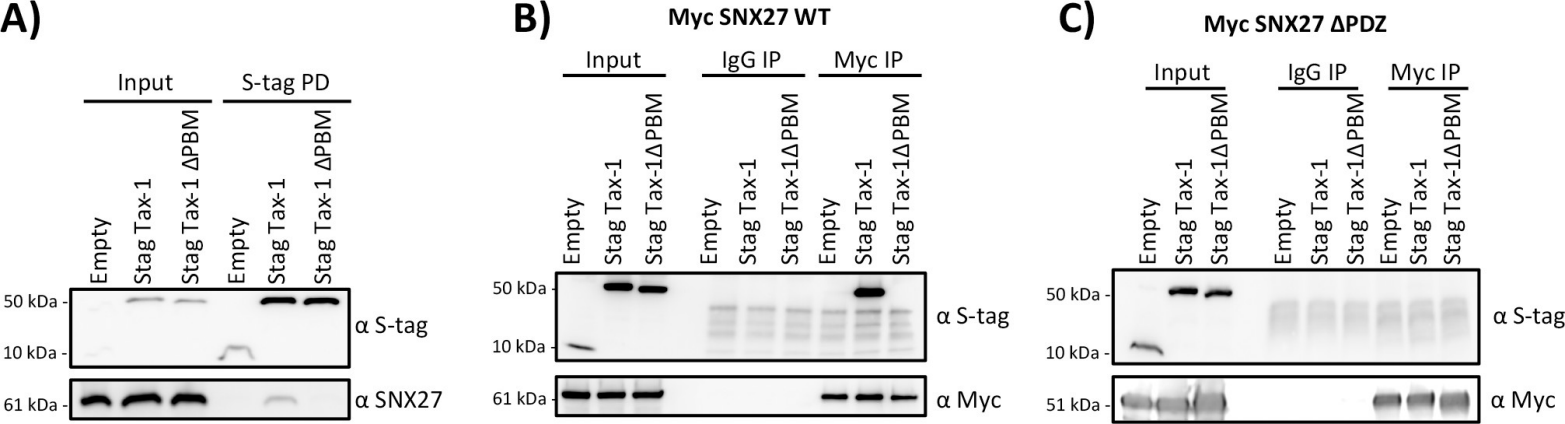
Tax-1 Interaction with SNX27 was Dependent on Tax-1 PBM and SNX27 PDZ Domains. **A**) S-tag pull-down demonstrating importance of Tax-1 PBM for interaction with SNX27. HEK293T cells were transfected with 10 μg of indicated S-tag construct (empty, Tax-1, or Tax-1 ΔPBM). 24 hours post transfection S-tag pull-downs were performed. Samples were subjected to SDS-PAGE followed by immunoblotting and probed for SNX27 and S-tag, as shown. **B and C)** Co-IP of SNX27 to demonstrate the importance of the SNX27 PDZ domain in the interaction of Tax-1 and SNX27. HEK293T cells were transfected with 5 μg either Myc-SNX27 WT (B) or Myc-SNX27 ΔPDZ (C) along with 5 μg of indicated S-tag constructs (empty, Tax-1, or Tax-1 ΔPBM). 24 hours post transfection, cells were collected, and lysates were subjected to co-IP using either Myc or normal IgG antibodies. Samples were subjected to SDS-PAGE followed by immunoblotting and probed for Myc and S-tag, as shown.

We next investigated which domains of Tax-1 and SNX27 are essential for their interaction. The mass spectrometry data demonstrated that SNX27 interacted with Tax-1 only when the PBM domain was present, and SNX27 is known to feature a PDZ domain (Fig 1C) [38]. To confirm that these domains were required for the interaction we again performed a S-tag pull-down using HEK293T cells transfected with either S-tag empty, S-tag Tax-1, or S-tag Tax-1 ΔPBM plasmid. Tax-1 was shown to interact with SNX27; however, Tax-1 ΔPBM did not co-precipitate SNX27, as shown by the lack of an SNX27 band in the S-tag Tax-1 ΔPBM pull down sample (Fig 2A). We then queried whether the PDZ domain of SNX27 was required for this interaction. HEK293T cells were transfected with either Myc-SNX27 or Myc-SNX27 ΔPDZ along with the indicated S-tag constructs (empty, Tax-1, or Tax-1 ΔPBM). IP was performed using a Myc antibody, which revealed efficient IP of both Myc-tagged wild-type SNX27 and SNX27 ΔPDZ (Fig 2B and 2C, respectively). The only interaction detected in these assays was between wild type Tax-1 and wild type SNX27 (Fig 2B). These results demonstrated that the interaction requires the PBM and PDZ domains of Tax-1 and SNX27, respectively.

### Tax-1 expression alters the cellular localization of GLUT1

**Fig 3:**
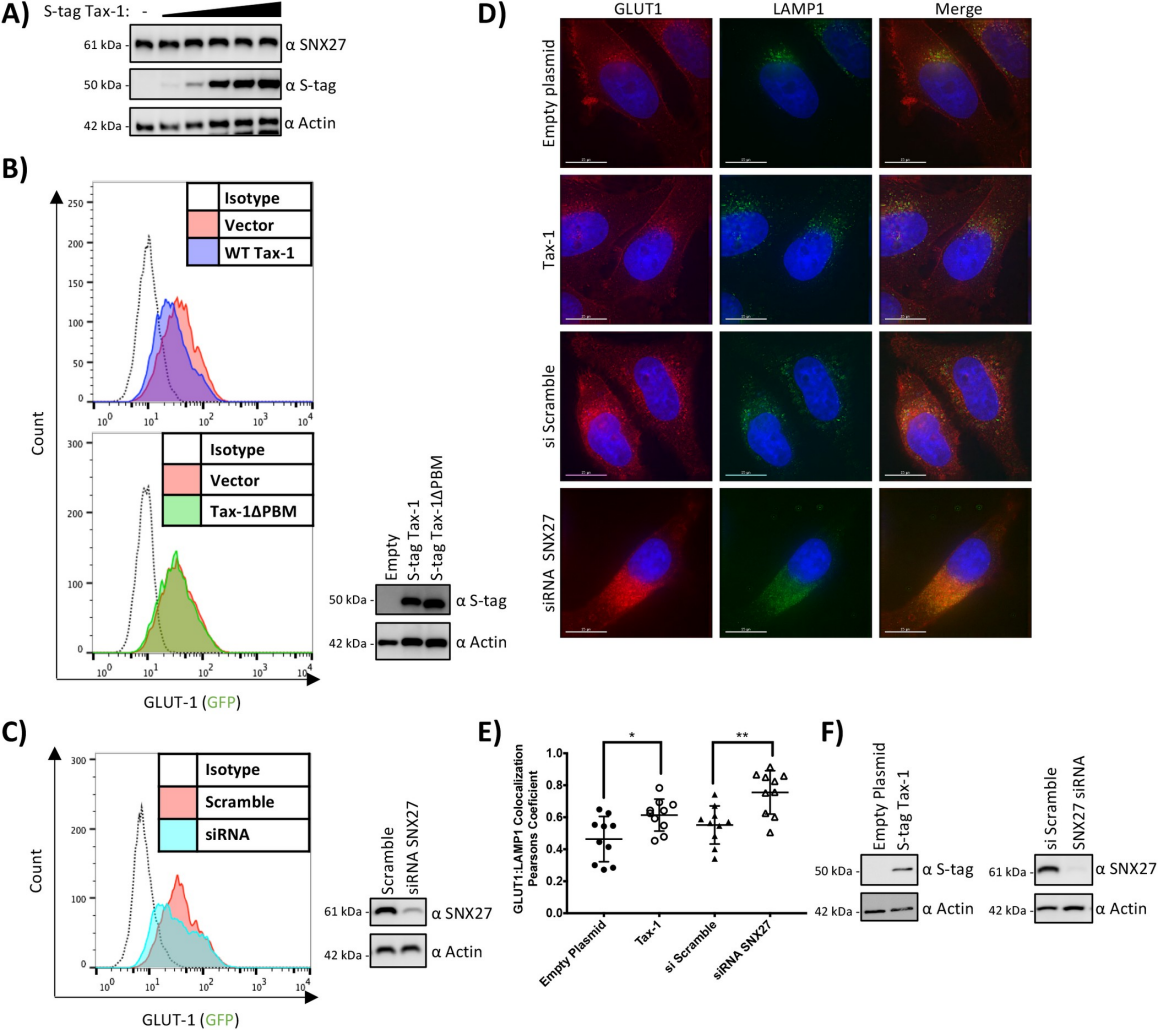
Tax-1 Expression altered the Cellular Localization of GLUT1. **A)** Tax-1 over-expression had no effect on SNX27 steady state levels. HEK293T cells were transfected with a titration of S-tag Tax-1 plasmid (100 ng to 2,000 ng). 24 hours post transfection cells were collected and lysed. Samples were measured for protein concentration and 10 μg of protein were from each sample were subjected to SDS-PAGE followed by immunoblotting. The membrane was probed for SNX27, S-tag, and Actin as shown. **B and C)** Tax-1 over-expression lowered the amount of GLUT1 located on the surface of cells. HEK293T cell were transfected with empty vector, S-tag Tax-1, or S-tag Tax-1 ΔPBM **(B)** or siRNA targeting SNX27 or a scramble control **(C)**. 24 hours post transfection cells were collected and stained with the GLUT1-RBD-GFP ligand per manufacturer’s instructions. Cells were then measured for GFP expression via flow cytometry. The histograms show the transfected cell populations with GFP intensity on the X-axis and number of cells on the Y-axis. A portion of the samples were lysed and subjected to SDS-PAGE followed by immunoblotting with the antibodies for SNX27, S-tag, or Actin, as indicated. **D)** Tax-1 over-expression increases colocalization of GLUT1 with LAMP1. Coverslips were seeded with HeLa cells. Cells were transfected with either empty plasmid, S-tag Tax-1, scramble siRNA, or siRNA against SNX27. 24 hours post transfection cells were fixed, permeabilized, and probed overnight with antibodies against GLUT1 and LAMP1. Alexaflour secondary antibodies were used. Coverslips were mounted on slides and deconvolution microscopy was performed using a DeltaVision microscope. Red represents GLUT1 staining, green represents LAMP1 staining, and blue represents DAPI staining. **E)** Calculated Pearson's coefficient of correlation for all conditions. Statistical analysis was performed using a one-way analysis of variance with Dunnett’s multiple comparison test. ** = p < 0.005, * = p <0.05. **F)** Western blot analysis of cells used in immunofluorescence assays. SNX27, S-tag, and Actin antibodies were used as indicated.

To understand what effect Tax-1 expression may have on SNX27, we first analyzed whether Tax-1 over-expression altered the steady state levels of SNX27. We transfected HEK293T cells with a titration of Tax-1 expression vector (from 100 ng to 2,000 ng), and lysates were subjected to immunoblotting. Our results indicate that over-expression of Tax-1 had no effect on SNX27 steady state levels, as shown by the probing with SNX27 antibody (Fig 3A).

As SNX27 is known to regulate the HTLV-1 receptor molecule GLUT1, we next set out to determine if Tax-1 expression had any effect on the ability of SNX27 to localize GLUT1 to the plasma membrane. HEK293T cells were transfected with S-tag empty, S-tag Tax-1, or S-tag Tax-1 ΔPBM expression plasmids. 24 hours post transfection, cells were collected and stained using the GLUT1-RBD-GFP ligand, which will specifically bind to GLUT1 on the surface of cells. Stained cells were then analyzed via flow cytometry. The cells transfected with S-tag Tax-1 showed lower amounts of surface GLUT1 compared to both the empty S-tag and the S-tag Tax-1 ΔPBM (Fig 3B). Immunoblotting was performed to confirm expression of the S-tag constructs (Fig 3B). HEK293T cells knocked down for SNX27 expression via siRNA were also tested for surface GLUT1 levels. A decrease in surface GLUT1 was detected when SNX27 was knocked down compared to the scramble control (Fig 3C). This data demonstrated that Tax-1 lowers the expression of GLUT1 on the plasma membrane, similar to how SNX27 knockdown lowers GLUT1 plasma membrane expression. Further, this function of Tax-1 was dependent on the PBM domain of Tax-1, which is required for the interaction between Tax-1 and SNX27.

It has been shown previously that when SNX27 is knocked down there is an increase in colocalization between GLUT1 and the lysosomal marker LAMP1, indicating an increase of GLUT1 in the lysosome prior to its potential degradation [20]. To determine if Tax-1 expression was causing an increase in colocalization between GLUT1 and LAMP1 we monitored their localization via immunofluorescence. HeLa cells were transfected with either Tax-1 expression plasmid or siRNA against SNX27. Cells were stained with antibodies for GLUT1 and LAMP1 and visualized using a deconvolution microscope. We observed the expected overlap of GLUT1 and LAMP1 when SNX27 was knocked down via siRNA (Fig 3D). We also saw a similar overlap when Tax-1 was expressed, while there was visibly less overlap in the siRNA control or empty plasmid transfected cells (Fig 3D). We then quantified the colocalization of LAMP1 and GLUT1 using the Pearson correlation coefficient (Fig 3E). We determined that both Tax-1 expression (p = 0.0134) and SNX27 knockdown using siRNA (p = 0.0022) caused a significant increase in colocalization of GLUT1 and LAMP1. Immunoblotting was performed to confirm the expression of Tax-1 and SNX27 in the HeLa cells (Fig 3F). This data demonstrates that Tax-1 expression resulted in an increase of GLUT1 and LAMP1 colocalization.

### SNX27 knockdowns increased HTLV-1 p19 Gag release

**Fig 4:**
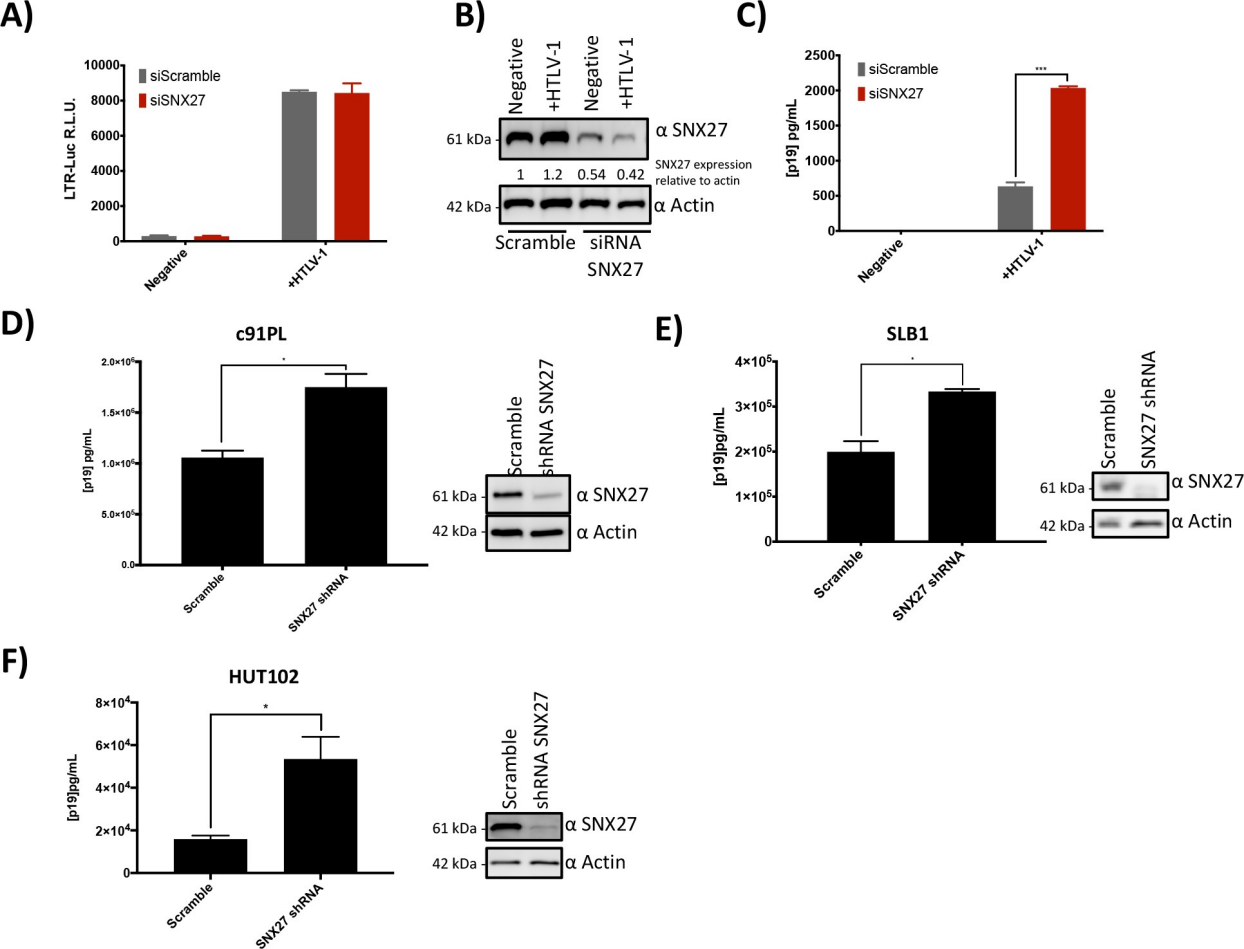
SNX27 Knockdown increased HTLV-1 p19 Gag Release. **A)** SNX27 knockdown did not alter LTR transactivation. HEK293T cells were transfected with siRNA against SNX27 or a scramble control. 24 hours post transfection cells were transfected again with the molecular clone of HTLV-1 and a LTR-Luc reporter plasmid. 48 hours after the second transfections cells were collected, lysed, and subjected to a luciferase assay. Samples in grey were transfected with siRNA scramble and samples in red were transfected with SNX27 siRNA. **B)** Immunoblot showing expression of SNX27 in samples from A and C. Lysates were subjected to SDS-PAGE and probed with SNX27 or Actin antibodies as indicated. **C)** SNX27 knockdowns increases p19 release into the supernatant. Supernatant was also collected from the samples in A and measured for p19 Gag (Matrix) via ELISA. **D-F)** The HTLV-1 producing cell lines c91PL (D), SLB-1 (E) and HUT-102 (F) were transduced with lentivirus expressing either scramble shRNA or shRNA targeting SNX27. Stable cells lines were generated via antibiotic selection. 1x10^6^ cells were seeded in 12-well dishes. 48 hours post seeding, supernatants and cells were collected. Supernatants were assayed for p19 via ELISA while cells were lysed and subjected to SDS-PAGE followed by immunoblotting for SNX27 and Actin as indicated. * = p < 0.05 and *** = p < 0.0005 as determined by Student's *t* test.

We next set out to discern any relevance of the Tax-1/SNX27 interaction to HTLV-1 biology. To rule out any downstream effects on the virus life cycle due to changes in Tax-1 transcriptional activity, we first analyzed whether knockdown of SNX27 affected Tax-1 LTR transcriptional activity. HEK293T cells were transfected with siRNA against SNX27 or scramble siRNA being used as a control. 24 hours post transfection with siRNA, cells were also transfected with the HTLV-1 full-length molecular clone, the LTR-luc reporter construct, and RTK as a transfection control. 48 hours later, supernatant and cells were collected. The cells were lysed and subjected to both immunoblot and Dual Glo luciferase assays. The luciferase assay results revealed no statistically significant difference in Tax-1 LTR activity between the scrambled or SNX27 knockdown cell lines (Scramble to SNX27 siRNA p = 0.4538) (Fig 4A). Immunoblotting revealed that SNX27 expression was reduced in the SNX27 knockdown samples, as expected (Fig 4B). Together, these data demonstrated that knock down of SNX27 did not alter Tax-1 transactivation of the HTLV-1 viral promoter.

We have demonstrated that Tax-1 expression changed the localization of the HTLV-1 receptor molecule GLUT1 within the cell. Previous literature has shown that changes in GLUT1 expression levels have a direct effect on HTLV-1 infectivity [39]. Based on this, we proposed that if SNX27 was knocked down in HTLV-1 producing cells, higher levels of virions would be released. To test this hypothesis, the concentration of p19 Gag (Matrix) in the supernatant from cells transfected with the HTLV-1 molecular clone in Fig 4A were measured using a p19 ELISA. We observed that when SNX27 was knocked down there was a significant increase in the amount of p19 released into the supernatant by these cells (p = 0.0009). In addition, the HTLV-1-transformed cell lines c91PL, SLB-1, and HUT-102 were knocked down for SNX27 using shRNA. Scramble and SNX27 knockdown cells were cultured for 48 hours, supernatant was collected, and p19 concentration was measured by ELISA. The c91PL (Fig 4D), SLB-1 (Fig 4E), and HUT-102 (Fig 4F) SNX27 knockdown lines showed a significant increase of p19 in the supernatant compared to the scramble control (p = 0.0216 for c91PL, p = 0.0159 for SLB-1, and p = 0.0376 for HUT102). Immunoblotting was performed to confirm knockdowns (Fig 4D through 4F). Together, these data suggested that SNX27 knockdown causes an increase in virion release into the supernatant.

### SNX27 knockdown decreased HTLV-1 infectivity

**Fig 5:**
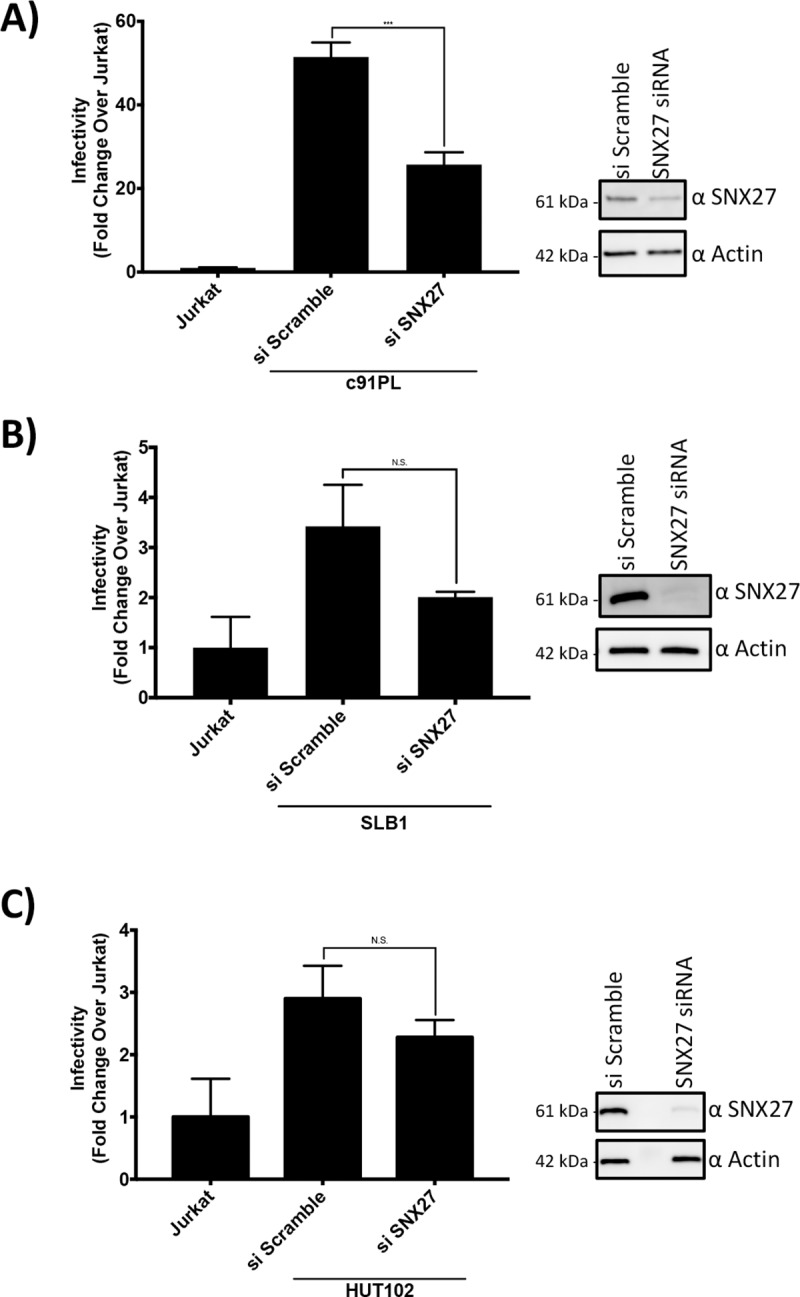
SNX27 Knockdown Lowered HTLV-1 Infectivity. **A-C)** SNX27 knockdown lowered HTLV-1 infectivity. c91PL (A), SLB-1 (B), or HUT-102 (C) cells were transfected with either scramble siRNA or SNX27 siRNA. 24 hours post transfection the cells were irradiated and then co-cultivated with Jurkat reporter cells. 24 hours post co-culture the cells were collected, lysed, and analyzed for luciferase activity. Lysates were also subjected to SDS-PAGE followed by immunoblotting for SNX27 or Actin as indicated. *** = p < 0.0005 and N.S. = p > 0.05 as determined by Student's *t* test.

Since SNX27 knockdown altered virus release, we hypothesized that HTLV-1 infectivity would also be altered by SNX27 knockdown. Using the Jurkat 18x21 Luc/RFP cell line we performed infectivity assays using the HTLV-1 producing cell lines c91PL, SLB-1, and HUT102. Producing cells were treated with siRNA against SNX27 or a scramble control, and then lethally irradiated. The irradiated cells were then co-cultured with the Jurkat reporter line for 24 hours (parental Jurkat cells were used as a negative control). Samples were then analyzed for luciferase levels, and a significant decrease in luciferase levels was observed in the c91PL cells knocked down for SNX27 expression (Fig 5A) (p = 0.0006). While the SLB-1 and HUT-102 samples did clearly show a downward trend in infectivity with SNX27 knocked down, it was not significant (Fig 5B and 5C) (p = 0.1391 for SLB-1 and p = 0.2782 for HUT-102). This data demonstrates that SNX27 knockdown in HTLV-1 producing cell lines lowers HTLV-1 infectivity.

## Discussion

HTLV-1 is the etiological agent of ATL, a malignancy of CD4^+^ T cells with a poor prognosis [4]. The closely related virus HTLV-2, is not associated with any human malignancies [7]. One of the key differences between the two viruses are the structural domains present on the regulatory protein Tax [40]. HTLV-1 Tax-1 features a PBM domain at the carboxyl terminus that HTLV-2 Tax-2 lacks. Several studies have identified the PBM domain as being important for the pathogenicity and persistence of HTLV-1 [15,17,41]. We set out to identify novel interacting proteins of Tax-1 that require the PBM domain. To achieve this, we performed pull down assays utilizing S-tag Tax-1 and S-tag Tax-1 ΔPBM. We then compared the interacting partners for the two Tax proteins to identify interactions dependent on the PBM. Our data set contained many known cellular interacting partners of Tax-1 and also demonstrated that interactions known to rely on the PBM were not detected with the PBM deleted mutant (Fig 1A).

We identified the Tax-1 interacting partner SNX27, a member of the sorting nexin family of proteins [18]. SNX27 is a unique member of the sorting nexin family due to the presence of a PDZ domain at the amino terminus of the protein [19]. Via the PDZ domain, SNX27 is able to bind to and regulate the localization and expression of its target proteins, including GLUT1 [20]. Previous studies have demonstrated that knockdowns of SNX27 result in a reduction of cell-surface localized GLUT1, and a decrease in the total levels of GLUT1 protein [20,21]. The cellular function of GLUT1 is to facilitate the transport of glucose across the plasma membrane, but GLUT1 also plays a role in the HTLV-1 viral life cycle. GLUT1 serves as one of three receptor molecules for HTLV-1, the others being HSPG and NRP-1, and is the receptor molecule required for virus fusion and entry into cells [23]. Another well studied retrovirus, HIV-1, has been shown to down regulate the expression of its receptor molecule (CD4) post infection [28]. In fact, HIV-1 has three known methods by which it lowers CD4 surface levels, and this down regulation is believed to benefit the virus in two ways [28]. First, it reduces the chances of a super infection event occurring, second it promotes more efficient virion production. The second benefit is thought to occur due to a decrease in interactions between the viral Envelope protein and CD4 at the plasma membrane of the infected cell. This interaction could result in less viral Envelope being available to the budding virions, leading to a decrease in infectious virion production or an increase in virions remaining attached to the cell. Thus, less CD4 at the plasma membrane increases HIV-1 virion production and infectivity. To date, HTLV-1 has no known mechanism for regulating receptor molecules post-infection, but recent studies have demonstrated that GLUT1 expression levels in HTLV-1 producing cells has an inverse relationship to the infectivity of VLPs produced from transfected HEK293T cells [27]. We proposed that the Tax-1 interaction with SNX27 may serve as the first known mechanism for HTLV-1 regulation of receptor molecules, with Tax-1 inhibiting the normal SNX27 function of recycling GLUT1 to the plasma membrane.

After confirming SNX27 and Tax-1 interact, and the PDZ and PBM domains of the two proteins, respectively, were required for this interaction (Figs 1 and 2), we next sought to analyze the significance of the SNX27 and Tax-1 interaction. We determined that any effects that Tax-1 expression may have on SNX27 function was not facilitated via expression level changes of SNX27 (Fig 3A). We next demonstrated that Tax-1 expression caused a decrease in GLUT1 on the surface of cells while the Tax-1 ΔPBM construct had no effect on surface GLUT1 levels (Fig 3B). This change in surface expression of GLUT1 was similar to the change seen when SNX27 expression was knocked down via siRNA (Fig 3C). This similarity, and the fact that the PBM of Tax-1 was required to see this change, suggests Tax-1 interaction with SNX27 results in the change of GLUT1 surface expression. Studies have demonstrated that knockdowns of SNX27 result in an increase of co-localization between GLUT1 and the lysosomal marker LAMP1. To determine if Tax-1 expression was causing a similar change in GLUT1/LAMP1 co-localization, we performed immunofluorescence analysis and found when Tax-1 was over-expressed there was an increase in colocalization similar to what was seen when SNX27 was knocked down via siRNA (Fig 3D and 3E). Together the flow cytometry and immunofluorescence data suggest that Tax-1 is inhibiting SNX27 function; decreasing GLUT1 surface expression and rerouting GLUT1 into lysosomes where it could potentially be degraded. While this study does not get to a mechanism as to how Tax-1 inhibits SNX27 function there are two distinct possibilities to consider. First, Tax-1 has been shown to mislocalize proteins it binds to via its PBM [31,35,42]. By mislocalizing SNX27, Tax-1 could block its normal function causing a decrease of GLUT1 on the cell surface. Second, Tax-1 has also been shown to competitively bind with cellular proteins blocking their normal interaction with other cellular proteins [43]. Tax-1 could block the interaction between SNX27 and GLUT1 resulting in the decrease of GLUT-1 on the cell surface.

Next, we analyzed what effect SNX27 expression levels had on HTLV-1 biology. We demonstrated that Tax-1 interaction with SNX27 has no effect on Tax-1 transactivation via an HTLV-1 LTR-luciferase based reporter assay. This result was expected as SNX27 is localized in the cytoplasm. Considering that previous studies have shown SNX27 knockdown results in lower levels of GLUT1 on the plasma membrane and changes in the GLUT1 expression level altered HTLV-1 infectivity, we set out to determine what effect SNX27 knockdowns would have on HTLV-1 virus release and infectivity. Using p19 Gag concentration in the supernatant as a surrogate for virus production, we found that SNX27 knock down in HEK293T cells transfected with the HTLV-1 molecular clone showed a significant increase of p19 concentration in the supernatant (Fig 4C). We also demonstrated that shRNA mediated knockdown of SNX27 in the HTLV-1 producing cell lines c91PL, SLB-1, and HUT-102 resulted in increases of p19 concentration in the supernatant (Fig 4D–4F). These results demonstrate that SNX27 regulation of GLUT1 on the surface of cells directly affects HTLV-1 virus release. We propose that this is due to less GLUT1 receptor molecule being available on the surface of cells, therefore allowing virions to escape the cell instead of being retained on the plasma membrane bound to GLUT1.

**Fig 6:**
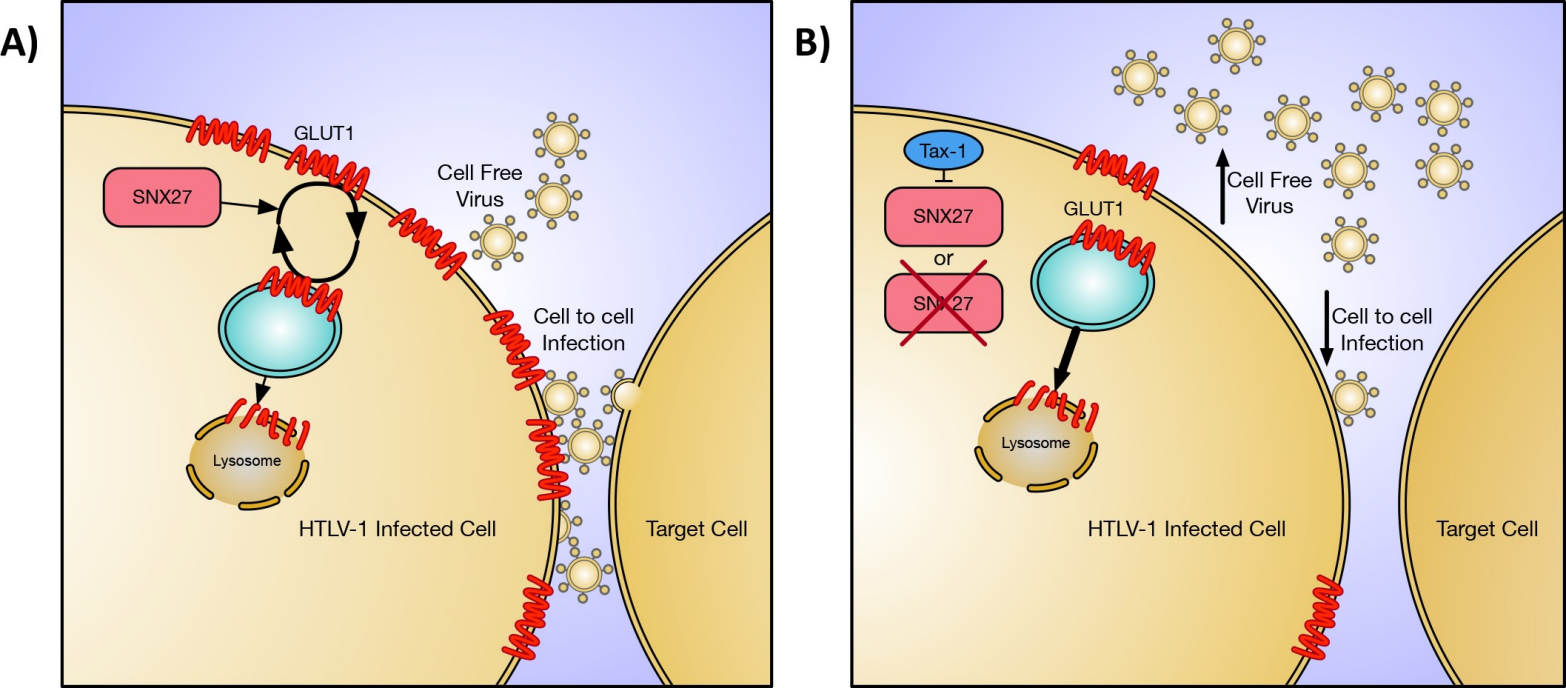
Proposed model for SNX27 role in HTLV-1 infectivity. **A)** Schematic diagram describing normal function of SNX27 promoting the recycling of GLUT1 to the plasma membrane. When GLUT1 is internalized into an endosomal vesical (shown in blue) SNX27 will bind to GLUT1 and return the vesical to the plasma membrane to maintain expression of GLUT1 on the cell surface. Some of these vesicles will progress further into the cell and fuse with a lysosome causing degradation of GLUT1. Normal HTLV-1 infection, which primarily occurs via cell to cell infection, is also shown. **B)** Schematic diagram demonstrating how loss of SNX27 function alters cell and HTLV-1 biology. SNX27 can be inhibited in two ways, by expression of Tax-1 blocking its function (top) or by knockdown of SNX27 expression (bottom). When SNX27 is inhibited less GLUT1 will be present at the surface of the cell. This decrease in GLUT1 results in an increase in HTLV-1 virus release into the supernatant and, consequently, a decrease in cell to cell mediated HTLV-1 infection.

We next measured the effect of SNX27 knockdown on HTLV-1 infectivity. Using a Jurkat reporter cell line, we found knockdown of SNX27 in HTLV-1 producing cell lines had an adverse effect on HTLV-1 infectivity (Fig 5). This result is counter to a previously published study that demonstrated decreases of GLUT-1 expression in virus producing cells resulted in more efficient infection [27]. This difference may be due to the experimental approaches used in both studies. The previous study utilized VLPs produced from transfected HEK293T cells, while we used complete HTLV-1 virus generated from HTLV-1 transformed T cell lines. Virus or VLPs produced from HEK293T may behave differently than virus produced from the more relevant T cell lines used in this study. Also, one should consider the expression level of GLUT1 on the surface of these different cell types. We have found that T cell lines express little detectable GLUT1 on the cell surface compared to HEK293T cells (S1 Fig). This difference in GLUT1 expression could alter the results found in the two studies. It is also interesting that HIV-1 infectivity increased when its receptor molecule was decreased on the surface of the producer cell [28,29]. We suspect this difference is due to the efficiency of cell-free infection of HIV-1 while HTLV-1 relies on cell-to-cell contact. HIV-1 benefits from virus being released into the supernatant as it is efficiently transmitted cell free *in vitro*. While HTLV-1 may depend on retaining the virion on the surface of the cell to facilitate cell-to-cell transmission. In Fig 6 we propose a model of how the interaction of SNX27 and Tax-1 modulates GLUT1 and HTLV-1 infectivity consistent with our data. Further work will be needed to determine the exact mechanism by which the Tax-1/SNX27 interaction is involved in modulating GLUT1 cellular localization and HTLV-1 infectivity.

In summary, we have discovered a novel interacting cellular partner of Tax-1, SNX27, and demonstrated that this interaction is mediated by the PBM of Tax-1 and the PDZ of SNX27. Furthermore, we show that Tax-1 is capable of altering GLUT1 localization, and promotes its transport to the lysosome for potential degradation. SNX27 knockdowns cause a significant increase in the number of virions present in the supernatant, and adversely affects cell-to-cell mediated HTLV-1 infectivity. To date, there is no known mechanism of HTLV-1 receptor molecule regulation post infection, and our work suggests that Tax-1 is capable of regulating GLUT1 localization through its interaction with SNX27. The identification of Tax-1 and SNX27 as regulators of HTLV-1 receptor molecules reveals new potential targets to combat viral spread. Future studies should clarify how Tax-1 alters the infectivity of the virus through SNX27 and determine the potential of SNX27 as a therapeutic target.

## Materials and methods

### Plasmids

The S-tag Tax-1 and S-tag Tax-1ΔPBM expression plasmids were generated via PCR amplification of the Tax gene from their respective HTLV-1 molecular clones generated previously [17,44]. PCR products were generated with NheI and XmaI restriction sites flanking the Tax-1 gene. The Tax-1 open reading frame was then inserted into the pTriEx-4 Neo vector (Novagen) in frame with the N-terminal 6xHis-S-Tag using the NheI and XmaI restriction sites. The LTR-luc reporter construct and TK-renilla control plasmid were described previously [45]. The Myc-SNX27 and Myc-SNX27 ΔPDZ expression vectors were a kind gift from Dr. M. Playford (National Heart, Blood, and Lung Institute. NIH. Bethesda, MD).

### Cell culture

All cells were maintained in humidified incubators at 37°C and 5% CO_2_. HEK293T (ATCC) and HeLa (ATCC) cells were maintained in DMEM supplemented with 10% FBS, 2 mM glutamine, penicillin (100 U/mL), and streptomycin (100 μg/mL). SLB-1 (generated previously [37]) cells were maintained in Iscove’s modified DMEM supplemented with 10% FBS, 2 mM glutamine, penicillin (100 U/mL), and streptomycin (100 μg/mL). HUT102 (ATCC), c91PL (a kind gift from Dr. Chou-Zen Giam), Jurkat (ATCC), and Jurkat 18×21 Luc/RFP (a kind gift from Dr. Chou-Zen Giam) reporter cell lines were maintained in RPMI 1640 supplemented with 10% FBS, 2 mM glutamine, penicillin (100 U/mL), and streptomycin (100 μg/mL).

### Pull-downs

S-tag pull-downs for mass spectrometry and interaction verification were performed as follows. Two 10-cm dishes were seeded with 2.5x10^6^ HEK293T cells, and 24 hours later transfected with 10 μg of indicated expression plasmid using TransIT-2020 (Mirus) per manufacturer’s instructions. 24 hours post-transfection, cells were collected in ice cold PBS and cell pellets from both plates were combined by centrifugation. Cells were lysed using passive lysis buffer (Promega) supplemented with cOmplete mini EDTA-free protease inhibitor (Roche). Lysates were cleared of debris via centrifugation and 10% of the lysate was retained as input. Lysates were then incubated with 40 μL of S-agarose (Novagen) overnight. Beads were spun down (300g for 4 minutes) and washed four times with RIPA lysis buffer. Beads were then resuspended in 1× SDS loading dye and boiled for 10 minutes. Samples were then loaded on an SDS-PAGE gel and either subjected to mass spectrometry or immunoblotting.

### Co-Immunoprecipitations

For HEK293T co-immunoprecipitations, a 10-cm dish was seeded with 2.5x10^6^ HEK293T cells. 24 hours later the cells were transfected with 10 μg of indicated plasmids using TransIT-2020 (Mirus) per manufacturer’s instructions. 24 hours post-transfection cells were collected and lysed in NP-40 lysis buffer (150mM NaCl, 50mM Tris-Cl pH 8.0, and 1% NP-40) supplemented with cOmplete mini EDTA-free protease inhibitor (Roche), and 10% of the lysates was retained as input. The remaining lysates were incubated overnight with indicated antibody. Antibodies used: anti-SNX27 (Abcam, 2 μg), anti-Myc (Abcam, 2 μg), rabbit anti-Tax-1 antisera (2 μL) [46], mouse normal IgG control (Santa Cruz, 2 μg), or rabbit normal IgG control (Santa Cruz, 2 μg). The following day, Dynabeads Protein G (Thermo Fisher) were added and samples were gently rocked for two hours. Beads were washed four times in NP40 lysis buffer via magnetic separation. Beads were resuspended in 1× SDS loading dye and boiled for 10 minutes. Supernatants were subjected to immunoblotting.

SLB-1 co-immunoprecipitations were performed as follows. 5x10^6^ SLB-1 cells were lysed in NP-40 lysis buffer supplemented with cOmplete mini EDTA-free protease inhibitor (Roche). 10% of the lysates was retained as input. The lysate was incubated overnight with SNX27 antibody (Abcam, 2 μg) or a mouse normal IgG control (Santa Cruz, 2 μg). The following day, Dynabeads Protein G (Thermo Fisher) were added and samples were gently rocked for two hours. Beads were washed four times in NP40 lysis buffer via magnetic separation. Beads were resuspended in 1× SDS loading dye and boiled for 10 minutes. Supernatants were subjected to immunoblotting.

### Mass spectrometry

S-tag pull-down samples were loaded and separated on Mini-PROTEAN TGX Precast 4%–20% SDS-PAGE gels (BioRad). The gel was then stained using GelCode Blue Stain Reagent (Thermo Fisher Scientific) per manufacturer’s instructions. Whole lanes were excised from the gel and subjected to proteomics analysis as described previously [47].

Capillary-liquid chromatography-tandem mass spectrometry (Capillary-LC/MS/MS) for global protein identification was performed on a Thermo Finnigan LTQ Orbitrap mass spectrometer equipped with a microspray source (Michrom Bioresources Inc.) operated in positive ion mode. Samples were separated on a capillary column (0.2x150 mm Magic C18AQ 3 μ 200A, Michrom Bioresources Inc.) using an UltiMateTM 3000 HPLC system (LC-Packings/Dionex Co.). The scan sequence of the mass spectrometer was based on the data-dependent TopTen method. The resolution of a full scan was set at 30,000 to achieve a high mass accuracy MS determination.

The RAW data files collected on the mass spectrometer were converted to mzXML and MGF files by use of MassMatrix data conversion tools (version 1.3, www.massmatrix.net/download). The resulting MGF files were searched using Mascot Daemon (Matrix Science version 2.2.2) and the database was searched against the full SwissProt database version 57.5 (471472 sequences; 167326533 residues) or NCBI database version 20091013 (9873339 sequences; 3367482728 residues). Considered modifications (variable) were methionine oxidation and the presence of carbamidomethyl cysteine. Three missed cleavages for the enzyme were permitted with a peptide tolerance of 1.2 Da, and the MS/MS ion tolerance was 0.8 Da. Search results were compiled and visualized using the Scaffold 4 software (Proteome Software). Unweighted spectrum count and percent coverage provided semi Squantitative data. Protein identifications were assigned using PeptideProphet (Institutes for Systems Biology). Proteins with 90% confidence were accepted with a minimum of one peptide displaying a 95% threshold confidence level.

### Immunoblotting

For whole cell lysate analysis, cells were lysed in NP-40 lysis buffer supplemented with cOmplete mini EDTA-free protease inhibitor (Roche). Lysate concentrations were quantified via Pierce BCA protein assay kit (Thermo Fisher) and analyzed with a FilterMax F5 Microplate Reader (Molecular Devices). Equal amounts of protein were loaded and separated on Mini-PROTEAN TGX Precast 4%–20% SDS-PAGE gels (BioRad) and transferred to nitrocellulose membranes (GE). For co-IPs and pull downs, samples were loaded and separated on Mini-PROTEAN TGX Precast 4%–20% SDS-PAGE gels (BioRad) and transferred to nitrocellulose membranes (GE). Membranes were blocked in 5% milk in PBST. Blots were incubated with indicated primary antibody overnight and with secondary antibodies for one hour. Primary antibodies used: anti-S-tag (Abcam, 1:1,000), anti-β-actin(Abcam, 1:10,000), rabbit anti-Tax-1 antisera (1:1,000) [46], anti-SNX27 (Abcam, 1:1,000), and anti-Myc (Abcam, 1:1,000). Secondary antibodies used: goat-anti-rabbit HRP (Promega, 1:5,000), goat-anti-mouse HRP (Promega, 1:5,000) and donkey-anti-goat HRP (Santa Cruz Biotechnology, 1:5,000). VeriBlot secondaries were used for co-IPs to avoid IgG detection: anti-mouse IgG VeriBlot for IP (Abcam, 1:1,000) and anti-rabbit IgG VeriBlot for IP (Abcam, 1:200). Membranes were developed using Pierce ECL Western Blotting Substrate (Thermo Fisher) and imaged using an Amersham Imager 600 (GE).

### SNX27 knockdowns

TRC lentiviral shRNA vectors targeting SNX27 and negative control shRNAs (pLKO.1) were purchased from Dharmacon (GE). Lentivirus was generated by transfecting HEK293T cells with lentiviral shRNA construct, an HIV Gag/Pol, and a VSV-G expression vector. Transfections were performed using Lipofectimine 2000 (Invitrogen) per manufacturer’s instructions. 72 hours post-transfection, supernatants were collected and filtered through a 0.45 μm filter. The filtered supernatants were concentrated by overlaying on a 1 mL 25% sucrose cushion, and then performing centrifugation at 28,000 RPM for 1.5 hours in a Sorvall SW41 swinging bucket rotor. Supernatant was discarded, and the virus pellet was resuspended in 200 μL of RPMI 1640 media by gentle shaking overnight. Cells were transduced with lentivirus as follows; 5x10^5^ cells were collected and resuspended in 100 μL fresh media, 50 μL of concentrated virus, and 8 μg/mL polybrene, and placed in a single well of a 96-well round bottom plate. The plate was spun at 2,000 x g for 2 hours. Post centrifugation, cells were kept at 37°C for 1 hour. Cells were then washed and plated in 24-well tissue culture dishes. 48 hours post-transduction, selection was performed by adding 1 μg/mL puromyocin to all wells. Lines that grew under selection were then tested for efficient knockdown of SNX27 via immunoblotting.

Transient knockdowns were achieved via siRNA using the siGENOME Human SNX27 (81609) siRNA SMARTpool purchased from Dharmacon (GE). siGENOME Non-Targeting siRNA #3 (GE) was used as a scramble control. siRNA was transfected into HEK293T and HeLa cells using TransIT-TKO transfection reagent (Mirus) per manufacturer’s instructions. siRNA was transfected into c91PL, SLB-1, and HUT102 cells via nucleofection (Amaxa Cell Line Nucleofector Kit V using program X-001). Knockdown efficiency was measured via immunoblotting.

### Reporter assays and p19 Gag ELISAs

The transactivation efficiency of Tax-1 was measured using the LTR-luc reporter plasmid. 5x10^5^ HEK293T cells (scramble control and SNX27 knocked down) were seeded in 6-well dishes. The next day, cells were transfected using TransIT-2020 (Mirus) with the following plasmids: wtHTLV-1 (1,000 ng), LTR-luc (100 ng), and RTK (10 ng). 48 hours post-transfection cells were collected and lysed in passive lysis buffer (Promega). Lysates were then subjected to the Dual-Glo Luciferase Assay (Promega) per manufacturer’s instructions, and luciferase values read on a FilterMax F5 Microplate Reader (Molecular Devices). Supernatants were also collected and the concentration of HTLV-1 p19 Gag was measured by p19 ELISA (Zeptometrix, Buffalo, NY) performed per manufacturer’s instructions.

### GLUT1 flow cytometry

To measure the surface presence of GLUT1, a flow cytometry-based assay was performed. 1x10^5^ cells were stained with 2 μL of the GLUT1-RBD-GFP ligand (Metafora Biosystems) for 20 minutes at 37°C. Stained cells were washed in 1× PBS containing 2% Fetal Bovine Serum (FBS) two times and then resuspended in the wash solution. Stained and unstained (negative control) cells were analyzed on a Guava EasyCyte mini (MilliporeSigma) flow cytometer instrument. Data was analyzed and plotted using FlowJo software (FlowJo, LLC).

### Immunofluorescence and deconvolution microscopy

HeLa cells were grown on coverslips prior to transfection with either Tax-1 or empty expression plasmid or SNX27 or scramble siRNA. 24 hours post-transfection the cells were fixed with 4% paraformaldehyde for 20 minutes at room temperature followed by permeabilization with 0.1% Triton X-100 for 10 minutes at room temperature. Cells were then blocked with 5% bovine serum albumin (BSA) in 1× PBS for 1 hour at room temperature. Cells were then treated with GLUT1 (Abcam, AB15309; 1:100) and LAMP1 (Abcam, AB25630; 1:300) primary antibodies diluted in a 2% BSA 1× PBS solution overnight at room temperature. Alexa Fluor 568 goat anti rabbit and Alexa Fluor 488 goat anti mouse secondary antibodies (Life Technologies; 1:500) were diluted in 2% BSA 1× PBS solution and incubated on the cells for 1 hour at room temperature. Coverslips were mounted onto slides using VECTASHIELD mounting medium with DAPI (Vector Laboratories) per manufacturer’s instructions. Deconvolution images were acquired at room temperature using an inverted DeltaVision microscope (GE) with an oil immersion 60× objective lens. Subsequent colocalization analysis to determine Pearson’s coefficient of correlation and processing of images were performed using the DeltaVision software (GE).

### HTLV-1 infectivity assays

HTLV-1 infectivity was measured using a Jurkat 18×21 Luc/RFP reporter (Jurkat reporter) cell line in the method described by Alais et Al. [48]. Briefly, the HTLV-1 producing cell lines c91PL, SLB-1, and HUT-102 were transfected with either scramble siRNA or siRNA targeting SNX27. 24 hours post transfection, producing cell lines were irradiated (77 Gy) using a Gammacell irradiator. Irradiated cell lines were then co-cultivated with Jurkat reporter cells at a ratio of 20,000 producing cells to 100,000 Jurkat reporter cells. 24 hours later, cells were collected and lysed in 50 μL passive lysis buffer (Promega). Lysates were then measured for protein concentration via Bradford reagent. 40 μL of lysate was used for luciferase assay as described by the manufacturer (Promega). Luciferase values were normalized to protein concentration and then shown as fold change over a negative control (parental Jurkat cells co-cultivated with the Jurkat reporter cells).

### Statistics

Statistical analyses were performed using GraphPad Prism 7 software (GraphPad Software) as indicated. Colocalization studies were analyzed using a one-way analysis of variance with Dunnett’s multiple comparison test. All other studies were analyzed by Student's *t* test Statistical significance was defined as p < 0.05 (* = p < 0.05, ** = p < 0.005, *** = p < 0.0005).

## Supporting information

S1 Fig GLUT1 surface expression not detectable on T Cells Compared to HEK293T. The indicated cells were collected and stained with the GLUT1-RBD-GFP ligand per manufacturer’s instructions. Cells were then measured for GFP expression via flow cytometry. The histograms show the cell populations with GFP intensity on the X-axis and number of cells on the Y-axis. Isotype stained cells are in red, while GLUT1-RBD-GFP stained cells are in blue. Cells analyzed: **A)** Resting CD4+ T cells, **B)** Activated CD4+ T cells, **C)** SLB-1 cell line, **D)** HUT-102 cell line, **E)** Jurkat cell line, and **F)** HEK293T cell line.
(TIF)
