# Supplementary figures and images for: HTLV-1 Tax-1 interacts with SNX27 to regulate cellular localization of the HTLV-1 receptor molecule, GLUT1
Source: PLoS One. 2019 Mar 21;14(3):e0214059. doi: 10.1371/journal.pone.0214059 (PMC6428263; doi:10.1371/journal.pone.0214059)

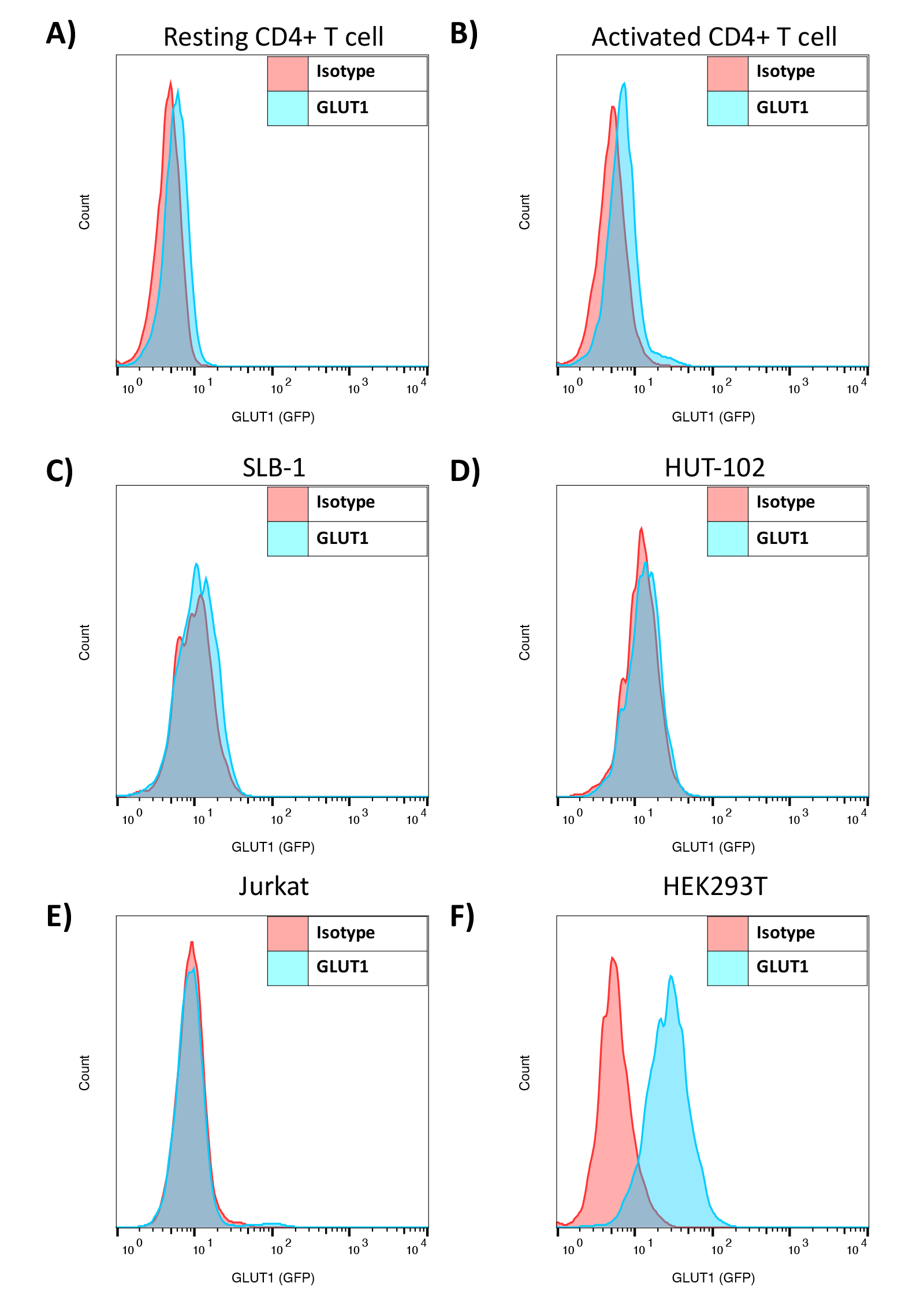

Supplement: S1 Fig — The indicated cells were collected and stained with the GLUT1-RBD-GFP ligand per manufacturer’s instructions. Cells were then measured for GFP expression via flow cytometry. The histograms show the cell populations with GFP intensity on the X-axis and number of cells on the Y-axis. Isotype stained cells are in red, while GLUT1-RBD-GFP stained cells are in blue. Cells analyzed: A) Resting CD4+ T cells, B) Activated CD4+ T cells, C) SLB-1 cell line, D) HUT-102 cell line, E) Jurkat cell line, and F) HEK293T cell line. (TIF) [file pone.0214059.s001.tif]
